# J. Francis Foltz, D.D.S.

**Published:** 1892-01

**Authors:** 


### J. FRANCIS FOLTZ, D.D.S.

DIED in East Boston, Mass., December 3, 1890, J. Francis Foltz, D.D.S., graduate of Boston Dental College, class of 1876.

The following resolutions were passed at the last annual meeting of the New England Dental Society, held in Boston, October 29 and 30, 1891:

WHEREAS, It has pleased our Heavenly Father to call from our midst a beloved brother, co-worker, and friend, Dr. J. Francis Foltz, of East Boston, Mass., therefore, be it

*Resolved*, That this Society loses an active member, whose faithful, earnest work is known throughout the profession.

*Resolved*, That this expression of our regret for his loss be entered upon the records of this Society, and that a copy of these resolutions be sent to his afflicted family and to the dental journals.

Signed: GEORGE F. CHENEY,
C. W. CLEMENT,
EDWARD B. DAVIS.

Attest: EDGAR O. KINSMAN, *Secretary.*

BOSTON, MASS., October 29, 1891.

